# Improving Care through Improv: Understanding Participant Experiences with an Innovative Training Program for Family and Friends of Persons Living with Dementia

**DOI:** 10.1002/alz70858_103952

**Published:** 2025-12-25

**Authors:** Candace Lynn Kemp, Jennifer Craft Morgan

**Affiliations:** ^1^ Georgia State University, Atlanta, GA, USA

## Abstract

**Background:**

Acquiring relevant skills and coping strategies is key to caregivers’ ability to succeed and remain in their roles with optimal well‐being, especially in the context of dementia. Quantitative analyses from a pilot test of “Improving Care through Improv,” an 8‐hour group‐based program combining foundational improvisational (improv) theatre skills with dementia‐care knowledge shows its preliminary efficacy for improving caregiving mastery and well‐being. Here, we extend these findings by seeking to: 1) examine care partner responses to the training, including the format, content, and delivery methods; and 2) understand care partners’ experiences enacting the training during interactions with their person living with dementia.

**Methods:**

The study involved 43 informal care partners of persons with moderate dementia. We present analysis of qualitative data collected as a complement to quantitative survey data. Our focus is on understanding caregivers’ subjective experiences through analysis of open‐ended survey responses, fieldnotes from training sessions (*n* = 16), and data from focus groups (*n* = 4).

**Result:**

Participants perceived the training as fulfilling a “need” by conveying “valuable” and “practical skills” in “fun, educational, and engaging” ways. The peer‐group format enhanced the experience. As the program progressed, most participants described experiencing shifts in their mindset and behaviors. They used newly acquired skills, including the “ability to be in the moment,” and “meet people where they are,” and felt that doing so improved care interactions and relationships and their ability to manage difficult situations.

**Conclusion:**

“Improving Care through Improv,” shows promise for increasing caregiver mastery and enhancing care relationships and warrants further investigation.

